# The Ercc*1*^-/Δ^ mouse model of accelerated senescence and aging for identification and testing of novel senotherapeutic interventions

**DOI:** 10.18632/aging.202321

**Published:** 2020-12-22

**Authors:** Alec Wong, Tra Kieu, Paul D. Robbins

**Affiliations:** 1 Institute on the Biology of Aging and Metabolism, Department of Biochemistry, Molecular Biology and Biophysics, University of Minnesota, Minneapolis, MN 55455, USA

**Keywords:** cellular senescence, aging, DNA repair, DNA damage, senolytic

## Abstract

Progeroid murine models represent an emerging tool to investigate mechanisms of aging in an expedient and efficient manner. One prominent mechanism of aging is the accumulation of DNA damage and subsequent increase in cellular senescence, leading to age related pathologies. *Ercc1^-/Δ^* hypomorphic mice, which have a reduced level of the ERCC1-XPF DNA repair endonuclease complex, accumulate spontaneously occurring endogenous DNA damage similar to naturally aged mice, but at a faster rate. The resulting genomic damage gives rise to a senescent cell burden that is comparable to that of a naturally aged mouse. In fact, the expression of senescence and senescence-associated secretory phenotype (SASP) markers in 4-5-month-old *Ercc1^-/Δ^* mice, along with other measurements of senescence, were equivalent and never exceeded the extent of that found in naturally aged mice. Furthermore, many features of both natural murine aging and human aging are present in *Ercc1^-/Δ^* mice. An emerging use of these mice is the ability to study age-related signaling pathways, including identifying different types of senescent cells and their key senescent cell anti-apoptotic pathways (SCAPs). Most importantly, this model represents a rapid, cost-effective mouse model for the evaluation *in vivo* of senolytic drugs and other gerotherapeutics.

Aging is the progressive decline in physiological functioning with increasing age, and multiple mechanisms attempt to explain such a phenomenon. The field of geroscience, defined as the intersection of basic aging biology, chronic disease, and health, is always seeking to develop new models of aging or to refine existing ones. The *Ercc1^-/Δ^* progeroid mouse model is not only valuable for studying basic fundamentals of aging, but also testing therapeutic interventions or evaluating surrogate biomarkers of biological age. These mice mimic the human progeroid syndrome XFE and age roughly six times faster than WT mice due to a deficiency in the ERCC1-XPF DNA repair endonuclease complex [1, 2]. DNA damage, in particular cyclopurine adducts (cPu), are known to increase in the multiple organs of mice with age and in *Ercc1^-/Δ^* [3]. These mice accumulate DNA damage like naturally aged mice, albeit at a faster rate and exhibit the onset of numerous aspects of both natural murine aging and human aging [1]. One mechanism for this progeroid phenotype in *Ercc1^-/Δ^* mice is that enhanced endogenous DNA damage promotes cellular senescence, which in turn drives aging [4–6].

The senescent cell burden is known to impair tissue homeostasis, enhance age-associated pathologies, and shorten both health span and lifespan [7–12]. Senescent cells can negatively impact the surrounding healthy tissue environment via the release of inflammatory soluble factors in what is known as the senescence-associated secretory phenotype (SASP) [13]. Multiple biomarkers are used to identify senescent cells both *in vitro* and *in vivo.* Consistent with prior findings*,* naturally aged mice display elevated senescence and SASP marker expression in multiple tissues including in peripheral T lymphocytes that are used for measuring biological age in humans. Remarkably, increased expression of senescence and SASP genes was found in those same tissues of 4-5-month-old *Ercc1^-/Δ^* progeroid mice, often to a comparable extent to, but never exceeding the level found in old WT mice. These findings were supported by the increase in percentage of cells positive for senescence-associated ß-galactosidase staining in multiple tissues of *Ercc1^-/Δ^* and old WT mice. Elevated levels of multiple SASP factors were present in the blood of both naturally aged mice and *Ercc1^-/Δ^* mice relative to their young littermate controls. The progressive increase in senescent cell burden was measured by cross-sectional and longitudinal predictor using both gene expression analysis and *in vivo* bioluminescence imaging of a p16^INK4a^-luciferase transgenic reporter [6]. These findings strongly support the notion that the *Ercc1^-/Δ^* mouse model of accelerated aging experiences a similar breadth and depth of senescent cell burden as do naturally aged mice, but which accumulates at an accelerated rate.

This model of accelerated senescence and aging can be used to further study the close relationship between senescent cells and aging with the intention of developing techniques for predicting health span and treating age-related disease. Due to the fact that there are tissue-specific senescence profiles in multiple tissues, which utilize different senescent cell anti-apoptotic pathways (SCAPs), no one senolytic will be pancellular. *Ercc1^-/Δ^* mice, which have a comparable profile of cellular senescence in comparison to naturally aged mice, can also be used to further study SCAPs and identify senolytics which are effective against specific tissue or cell populations. In fact, many senolytics have already been tested in *Ercc1^-/Δ^* mice [14–16]. The natural product fisetin has been shown as an effective senotherapeutic in both mice and human cells. Treatment of aged WT and *Ercc1^-/Δ^* mice with fisetin reduced expression of senescence markers in multiple tissues leading to reduced age-related pathology, and an overall health span improvement [15]. Altogether, the results demonstrate that *Ercc1^-/Δ^* mice are a proper model of accelerated aging, allowing for efficient, cost effective experimentation and evaluation of senotherapeutics for aging studies. Utilization of these mice should accelerate the identification of new therapeutic targets and the development of age-related interventions.
